# Supplementary material for: Genome-Wide DNA Methylation Differences between Bos indicus and Bos taurus
Source: Animals (Basel). 2023 Jan 5;13(2):203. doi: 10.3390/ani13020203 (PMC9854497; doi:10.3390/ani13020203)
Supplement: Supplementary file 1 [file animals-13-00203-s001.zip › animals-2082736-supplementary.pdf]

Contents

Table S1. Summary of WGBS data and comparison 1
Table S2. Samples DNA methylation information statistics 2
Table S3. GO enrichment results of common differential methylation genes in spleen and liver groups 3
Table S4. KEGG enrichment results of common differential methylation genes in spleen and liver groups 13
Figure S1. PBI15 whole-genome DNA methylation patterns 15
Figure S2. PBI52 whole-genome DNA methylation patterns 16
Figure S3. PBT24 whole-genome DNA methylation patterns 17
Figure S4. PBT47 whole-genome DNA methylation patterns 18
Figure S5. PBT57 whole-genome DNA methylation patterns 19
Figure S6. GBI52 whole-genome DNA methylation patterns 20
Figure S7. GBT47 whole-genome DNA methylation patterns 21
Figure S8. GBT57 whole-genome DNA methylation patterns 22

Table S1. Summary of WGBS data and comparison

Samples	Raw Reads	Clean reads	Mapped Ratio/%	Unique Mapped reads	Unique Mapped Ratio/%
PBI15	612,301,364	602,868,002	70.05	404,287,138	67.1
PBI33	650,969,276	626,268,002	67.1	403,538,174	64.4
PBI52	669,159,636	617,256,002	65.97	390,146,476	63.2
PBT24	647,750,984	619,194,002	71.06	422,818,734	68.3
PBT47	663,917,898	618,738,002	70.26	415,068,294	67.1
PBT57	661,222,082	617,742,002	69.77	412,076,758	66.7
GBI33	801,939,510	639,846,710	77.76	474,240,974	73.4
GBI52	1,052,516,242	581,479,484	74.39	412,121,624	70.7
GBT47	1,047,965,290	585,907,216	78.21	436,516,690	74.6
GBT57	1,054,457,492	601,346,728	75.76	435,023,082	73.3

Table S2. Samples DNA methylation information statistics

Sample	mC	mCG	mCHG	mCHH	mCG Ratio/%	mCHG Ratio/%	mCHH Ratio/%
PBI15	11,522,713	11,104,675	94,438	323,600	96.37	0.81	2.80
PBI33	13,200,669	12,716,368	111,495	372,806	96.33	0.84	2.82
PBI52	13,395,589	12,911,448	109,985	374,156	96.38	0.82	2.79
PBT24	14,151,212	13,542,250	141,578	467,384	95.69	1.00	3.30
PBT47	17,023,754	16,298,797	165,178	559,779	95.74	0.97	3.28
PBT57	15,836,547	15,151,162	152,229	533,156	95.67	0.96	3.36
GBI33	16,764,297	16,069,376	152,432	542,489	95.85	0.91	3.24
GBI52	15,062,634	14,400,650	137,083	524,901	95.61	0.91	3.48
GBT47	14,718,503	14,027,871	144,241	54,391	95.31	0.98	3.71
GBT57	13,134,721	12,471,623	136,261	526,837	94.95	1.04	4.01

Table S3. GO enrichment results of common differential methylation genes in spleen and liver groups

Term	p-value	Number of genes	Genes
GO:1902578~single-organism localization	4.63E-05	59	DPP10, ERO1A, RAB3C, KCNC2, GRM1, SYNE2, PPP3CA, GRIP1, LIPC, NETO1, PIP4K2A, SLC16A7, EPG5, KCNH1, UNC13C, CADPS2, KCNH7, TRPC4, PRKCE, SLC2A13, VPS13C, VPS13D, SLC6A11, WDR72, FRMD4A, PEX1, DNM3, VAMP7, AKAP9, SLC9A9, ELMO1, EPHA3, ABCG2, RAPGEF4, COLEC12, STK39, ATP2C1, GRIN2A, SV2C, SCFD2, GPC3, STXBP5, KCNN2, CACNG3, CORIN, CLASP1, SNCA, SLC38A4, ATP8B4, MICAL3, EXOC6B, MYO5A, BTBD9, GRIN2B, CENPE, GRIN3A, NEDD4, SLC26A7
GO:0032879~regulation of localization	8.90E-05	54	SEMA5A, DPP10, ERO1A, RAB3C, PTPRR, DOCK4, KCNC2, DOCK8, LDB2, SYNE2, PPP3CA, NIPBL, BMPER, NETO1, PRKG1, KCNH1, CADPS2, CASR, CEP135, PRKCE, PDE4D, CACNA2D1, ANO6, FRMD4A, VAMP7, NRG3, AKAP9, CDH13, MAPRE2, EPHA3, RAPGEF4, CAMK2D, STK39, NEDD9, ATP2C1, HDAC9, PCDH17, MGAT5, KRIT1, GPC3, STXBP5, GPC5, CTNNA3, CACNG3, CORIN, CLASP1, JAM2, SNCA, BTBD9, TF, GRIN3A, NEDD4, CNTN1, SMAP1
GO:0051641~cellular localization	4.33E-04	55	DPP10, ERO1A, RAB3C, MCM9, ITSN1, GPHN, SYNE2, PPP3CA, GRIP1, NIPBL, CCDC91, NETO1, PIP4K2A, EPG5, BBS9, TBC1D22A, ANKS1B, UNC13C, CADPS2, GRID2, PRKCE, VPS13C, VPS13D, ATRX, WDR72, PEX1, PARD3B, DNM3, VAMP7, AKAP9, CDH13, MAPRE2, EPHA3, RAP1GDS1, ATP2C1, PCDH17, TM9SF3, GRIN2A, WRN, SCFD2, GPC3, STXBP5, GPC5, CACNG3, CLASP1, CNTLN, SNCA, EXOC6B, MYO5A, BTBD9, GRIN2B, CENPE, GRIN3A, NEDD4
GO:0044707~single-multicellular organism process	7.27E-04	95	SEMA5A, ROBO2, PIWIL2, PTPRR, DOCK4, CTNND2, ZBTB20, LDB2, FRY, LIPA, SYNE2, PHEX, PREX2, PPP3CA, GRIP1, NIPBL, LIPC, BMPER, NETO1, CHL1, SCMH1, PIP4K2A, PLCE1, SVEP1, DIP2B, PRKG1, TP63, SOX5, GRID2, CASR, PCYT1B, DCC, TRPC4, PRKCE, PDE4D, CACNA2D1, ATRX, TCF12, TET2, ANO6, WDR72, VAMP7, MMP16, NRG3, PALLD, BANK1, TNMD, AKAP9, CDH13, RBM20, MAPRE2, EPHA3, COL15A1, CAMK2D, RNF180, CEP85L, GREB1L, NRXN3, PCDH15, STK39, HDAC9, PCDH17, RAD54B, SCEL, GRIN2A, WRN, CALD1, KRIT1, GPC3, SLIT3, CTNNA3, CSMD3, CACNG3, CLASP1, SNCA, GRXCR1, NEGR1, PLEKHA5, KLHL1, BTBD9, CNOT4, TF, PTPRB, CDK6, DAB1, GRIN3A, NEDD4, UST, CNTN1, SLC26A7, MNAT1, SMAP1, ADGRL3, FBN1
GO:0051234~establishment of localization	8.49E-04	80	DPP10, ERO1A, RAB3C, SLC44A1, KCNC2, ITSN1, GRM1, SYNE2, PPP3CA, GRIP1, NIPBL, LIPC, CCDC91, NETO1, PIP4K2A, SLC16A7, EPG5, TBC1D22A, KCNH1, UNC13C, CADPS2, CASR, CEP135, KCNH7, TRPC4, PRKCE, SLC2A13, VPS13C, CACNA2D1, VPS13D, SLC6A11, ANO6, SORCS1, FRMD4A, PEX1, PARD3B, DNM3, VAMP7, AKAP9, SLC9A9, ELMO1, CDH13, MICU3, EPHA3, ABCG2, RAPGEF4, COLEC12, SLC20A2, SLC41A2, STK39, ATP2C1, PCDH17, GRIN2A, SV2C, SCFD2, GPC3, STXBP5, KCNN2, CACNG3, CORIN, CLASP1, SNCA, SLC38A4, ATP8B4, GABRA3, MICAL3, EXOC6B, MYO5A, BTBD9, GRIN2B, LRP1B, CENPE, TF, GRIN3A, NEDD4, CACHD1, CNTN1, SLC26A7, SMAP1
GO:0040012~regulation of locomotion	9.66E-04	25	SEMA5A, ROBO2, PTPRR, DOCK4, DOCK8, STK39, NEDD9, LDB2, HDAC9, SYNE2, PPP3CA, NIPBL, BMPER, MGAT5, KRIT1, PRKG1, JAM2, CLASP1, SNCA, PRKCE, ANO6, TF, NRG3, CDH13, MAPRE2

GO:0040017~positive regulation of locomotion	0.001044264	17	SEMA5A, DOCK4, PRKCE, DOCK8, STK39, NEDD9, ANO6, HDAC9, SYNE2, PPP3CA, TF, NIPBL, MGAT5, CDH13, MAPRE2, CLASP1, JAM2

Term	p-value	Number of genes	Genes
GO:0044763~single-organism cellular process	0.001286407	168	SEMA5A, ERO1A, RAB3C, DGKB, CTNND2, ZBTB20, FRY, LIPA, PREX2, GRIP1, LIPC, BMPER, LIPH, CSPP1, PIP4K2A, PLCE1, DIP2B, TP63, KCNH1, SOX5, UNC13C, CADPS2, CASR, CEP135, KCNH7, PRKCE, CACNA2D1, WDR72, FRMD4A, FRMD4B, LEMD3, TBL1XR1, AKAP9, SLC9A9, COL4A6, ELMO1, COL4A5, UTRN, EPHA3, ABCG2, CDS1, ARHGEF28, CEP85L, STK39, PLD1, SAMSN1, FHIT, PCDH17, GRIN2A, CALD1, SCFD2, STXBP5, ST8SIA6, KCNN2, COL28A1, GRXCR1, ATP8B4, MICAL3, EXOC6B, MYO5A, GRIN2B, CNOT4, COQ3, DIAPH2, TF, CDK6, DAB1, GRIN3A, KIF4A, TMEM117, GNAQ, UST, CNTN1, MNAT1, CDK14, FBN1, DPP10, ROBO2, PIWIL2, PTPRR, DOCK4, KCNC2, DOCK8, GRIK1, PIK3C2G, LDB2, GRM1, SYNE2, GPHN, ADAMTSL1, PPP3CA, NIPBL, NETO1, MECOM, CHL1, GRM8, SPIN1, BBS9, EPG5, DLGAP1, PRKG1, VAV3, GRID2, PCYT1B, DCC, TRPC4, PDE4D, VPS13C, VPS13D, TCF12, ATRX, TET2, ANO6, HAUS6, PEX1, PARD3B, DNM3, VAMP7, MMP16, NRG3, BANK1, PALLD, TNMD, DPYD, CDH13, MICU3, CFAP54, PDZRN3, MAPRE2, RBMS3, RAPGEF4, COLEC12, COL15A1, NEDD9, HSD17B11, ATP2C1, HDAC9, RAD54B, SCEL, WRN, SV2C, MGAT5, GPC3, KRIT1, GPC5, SLIT3, CTNNA3, CSMD3, UGGT2, CACNG3, CLASP1, JAM2, SNCA, USP25, NEGR1, PLCL1, B3GAT2, GABRA3, KLHL1, AIG1, DYTN, CENPE, FMNL2, NEDD4, SMAP1, ADGRL3
GO:0016043~cellular component organization	0.001601882	107	SEMA5A, ERO1A, RAB3C, CTNND2, FRY, LIPA, PREX2, GRIP1, LIPC, SCMH1, PIP4K2A, PLCE1, DIP2B, TP63, UNC13C, CEP135, PRKCE, WDR72, LEMD3, TBL1XR1, AKAP9, COL4A6, ELMO1, COL4A5, EPHA3, ARHGEF28, PLD1, PCDH17, WDR70, CALD1, STXBP5, CNTLN, COL28A1, GRXCR1, ATP8B4, XRCC6, MICAL3, EXOC6B, MYO5A, BTBD9, PKIB, DIAPH2, TF, GRIN3A, KIF4A, UST, CNTN1, MNAT1, DPP10, ROBO2, PIWIL2, MCM9, SYNE2, GPHN, CLYBL, ADAMTSL1, PPP3CA, NIPBL, CHL1, BBS9, EPG5, PRKG1, VAV3, GRID2, DCC, VPS13C, VPS13D, ATRX, TET2, ANO6, HAUS6, PEX1, PARD3B, DNM3, VAMP7, MMP16, PALLD, TNMD, CDH13, CFAP54, PDZRN3, MAPRE2, COLEC12, COL15A1, NEDD9, ATP2C1, HDAC9, RAD54B, WRN, GPC3, CLVS2, SLIT3, CTNNA3, CSMD3, CLASP1, SNCA, NEGR1, KLHL1, YLPM1, CENPE, FMNL2, CENPI, NEDD4, EIF3H, SMAP1, ADGRL3
GO:0051674~localization of cell	0.002007111	35	SEMA5A, PTPRR, DOCK4, DOCK8, CEP85L, STK39, NEDD9, PIK3C2G, LDB2, PLD1, HDAC9, SYNE2, PPP3CA, NIPBL, BMPER, CHL1, MGAT5, GPC3, KRIT1, GPC5, CTNNA3, PRKG1, JAM2, CLASP1, DCC, PRKCE, ANO6, TF, DAB1, FMNL2, NRG3, ELMO1, CDH13, MAPRE2, ADGRL3
GO:0048870~cell motility	0.002007111	35	SEMA5A, PTPRR, DOCK4, DOCK8, CEP85L, STK39, NEDD9, PIK3C2G, LDB2, PLD1, HDAC9, SYNE2, PPP3CA, NIPBL, BMPER, CHL1, MGAT5, GPC3, KRIT1, GPC5, CTNNA3, PRKG1, JAM2, CLASP1, DCC, PRKCE, ANO6, TF, DAB1, FMNL2, NRG3, ELMO1, CDH13, MAPRE2, ADGRL3
GO:0044700~single organism signaling	0.002491584	94	SEMA5A, ERO1A, PTPRR, DOCK4, CAB39L, DGKB, CTNND2, DOCK8, ITSN1, GRIK1, PIK3C2G, GRM1, PREX2, PPP3CA, BMPER, NETO1, MECOM, GRM8, SPIN1, PIP4K2A, EPG5, PLCE1, SVEP1, DLGAP1, PRKG1, RGS6, TP63, RALGPS1, KCNH1, RGS7, ANKS1B, VAV3, UNC13C, CADPS2, GRID2, CASR, PRKCE, PDE4D, CACNA2D1, ATRX,

			ANO6, LEMD3, ITFG1, VAMP7, NRG3, TBL1XR1, BANK1, TNMD, AKAP9, COL4A6, ELMO1, CDH13, COL4A5, UTRN, MAPRE2, EPHA3, RAPGEF4, RBMS3, COLEC12, ARHGEF28, STK39, NEDD9, UBR1, ATP2C1, PLD1, FHIT, PCDH17, SCEL, GRIN2A, SV2C, MGAT5, GPC3, STXBP5, SLIT3, GPC5, CTNNA3, CACNG3, SNCA, PLCL1, GABRA3, GRIN2B, GPR137C, DYTN, TF, GRIN3A, NEDD4, TMEM117, GNAQ, GNB4, CNTN1, CDK14, ADGRL3, FBN1
GO:0007154~cell communication	0.002745912	95	SEMA5A, ERO1A, PTPRR, DOCK4, CAB39L, DGKB, CTNND2, DOCK8, ITSN1, GRIK1, PIK3C2G, GRM1, PREX2, PPP3CA, BMPER, NETO1, MECOM, GRM8, SPIN1, PIP4K2A, EPG5, PLCE1, SVEP1, DLGAP1, PRKG1, RGS6, TP63, RALGPS1, KCNH1, RGS7, ANKS1B, VAV3, UNC13C, CADPS2, GRID2, CASR, PRKCE, PDE4D, ATRX, ANO6, LEMD3, ITFG1, VAMP7, NRG3, TBL1XR1, BANK1, TNMD, AKAP9, COL4A6, ELMO1, CDH13, COL4A5, UTRN, MAPRE2, EPHA3, RAPGEF4, RBMS3, COLEC12, ARHGEF28, STK39, NEDD9, UBR1, ATP2C1, PLD1, FHIT, PCDH17, SCEL, GRIN2A, WRN, SV2C, MGAT5, GPC3, STXBP5, SLIT3, GPC5, CTNNA3, CACNG3, SNCA, PLCL1, GABRA3, BTBD9, GRIN2B, GPR137C, DYTN, TF, GRIN3A, NEDD4, TMEM117, GNAQ, GNB4, CNTN1, CDK14, ADGRL3, FBN1
GO:0007155~cell adhesion	0.003435718	29	ROBO2, CTNND2, DOCK8, NRXN3, PCDH15, NEDD9, ATP2C1, LPP, PCDH17, PPP3CA, CHL1, CTNNA3, TINAG, PRKG1, JAM2, CLASP1, COL28A1, GRID2, NEGR1, PRKCE, PARD3B, CDK6, PALLD, CNTN1, CDH13, UTRN, EPHA3, FBN1, ADGRL3

**Term**	**p-value**	**Number of genes**	**Genes**
GO:0065008~regulation of biological quality	0.006495337	64	SEMA5A, ERO1A, DOCK4, ZBTB20, LDB2, LIPA, GPHN, GRM1, PPP3CA, LIPC, NETO1, DIP2B, PRKG1, TP63, KCNH1, VAV3, UNC13C, CADPS2, GRID2, CASR, KCNH7, TRPC4, PRKCE, VPS13C, CACNA2D1, VPS13D, ATRX, SLC6A11, ANO6, DNM3, VAMP7, AKAP9, SLC9A9, MICU3, RAPGEF4, STK39, HSD17B11, ATP2C1, HDAC9, GRIN2A, WRN, KRIT1, STXBP5, CTNNA3, CACNG3, CORIN, SNCA, ATP8B4, XRCC6, GABRA3, YLPM1, BTBD9, GRIN2B, PKIB, TF, CDK6, GRIN3A, FMNL2, NBEA, NEDD4, GNAQ, SMAP1, FBN1
GO:0023051~regulation of signaling	0.006705528	56	PTPRR, DGKB, CTNND2, GRIK1, GRM1, PPP3CA, BMPER, NETO1, MECOM, GRM8, SPIN1, PIP4K2A, PLCE1, DLGAP1, RGS6, TP63, RALGPS1, RGS7, UNC13C, GRID2, PRKCE, PDE4D, LEMD3, VAMP7, NRG3, TBL1XR1, BANK1, TNMD, AKAP9, CDH13, MAPRE2, RBMS3, ARHGEF28, STK39, UBR1, ATP2C1, PCDH17, SCEL, GRIN2A, MGAT5, GPC3, STXBP5, SLIT3, GPC5, CACNG3, SNCA, PLCL1, BTBD9, GRIN2B, GPR137C, GRIN3A, NEDD4, GNAQ, CDK14, FBN1
GO:0007626~locomotory behavior	0.008807549	8	PREX2, NEGR1, CHL1, PRKCE, KLHL1, BTBD9, GRM1, SNCA
GO:0007165~signal transduction	0.013289333	84	SEMA5A, ERO1A, PTPRR, DOCK4, CAB39L, DGKB, CTNND2, DOCK8, ITSN1, PIK3C2G, GRM1, PREX2, PPP3CA, BMPER, NETO1, MECOM, GRM8, SPIN1, PIP4K2A, EPG5, PLCE1, SVEP1, DLGAP1, PRKG1, RGS6, TP63, RALGPS1, KCNH1, RGS7, ANKS1B, VAV3, UNC13C, GRID2, CASR, PRKCE, PDE4D, ATRX, ANO6, LEMD3, ITFG1, NRG3, TBL1XR1, BANK1, TNMD, AKAP9, COL4A6, ELMO1, CDH13, COL4A5, MAPRE2, EPHA3, RAPGEF4, RBMS3, COLEC12, ARHGEF28, STK39, NEDD9, UBR1, ATP2C1, PLD1, FHIT, SCEL, GRIN2A, MGAT5, GPC3, SLIT3, GPC5, CACNG3, SNCA, PLCL1, GABRA3, GRIN2B, GPR137C, TF, GRIN3A, NEDD4, TMEM117, GNAQ, GNB4, CNTN1, CDK14, ADGRL3, FBN1
GO:0048522~positive regulation of cellular process	0.016198832	80	SEMA5A, DPP10, ROBO2, PIWIL2, DOCK4, DOCK8, ZBTB20, LDB2, GRM1, SYNE2, PPP3CA, NIPBL, BMPER, NETO1, CSPP1, SPIN1, PIP4K2A, PLCE1, DIP2B, TP63, SOX5, VAV3, CADPS2, GRID2, CASR, CEP135, PRKCE, SLC2A13, VPS13C, VPS13D, ATRX, TCF12, TET2, ANO6, FRMD4A, PPP4R3B, VAMP7, TBL1XR1, BANK1, TFEC, AKAP9, CDH13,

			RBM20, MAPRE2, EPHA3, CREB5, COLEC12, CDS1, PARM1, RNF180, STK39, NEDD9, ATP2C1, PLD1, HDAC9, SCEL, GRIN2A, WRN, MGAT5, GPC3, TASP1, STXBP5, GPC5, CACNG3, CLASP1, JAM2, SNCA, XRCC6, NEGR1, GRIN2B, GPR137C, CENPE, PKIB, TF, CDK6, NEDD4, CNTN1, SMAP1, FBN1
GO:0044708~single-organism behavior	0.01792635	11	PREX2, GRIN2A, NETO1, CHL1, PRKCE, RNF180, KLHL1, BTBD9, PCDH17, SNCA
GO:0003008~system process	0.021785064	31	DOCK4, CAMK2D, STK39, PCDH15, GRM1, PPP3CA, NIPBL, GRIN2A, NETO1, CHL1, CALD1, CTNNA3, SVEP1, CACNG3, CORIN, IMPG1, PRKG1, GRXCR1, CASR, GRID2, MYBPC1, PDE4D, CACNA2D1, GABRA3, LHFPL3, BTBD9, GRIN3A, AKAP9, SLC26A7, ABCG2
GO:0048856~anatomical structure development	0.031076458	80	SEMA5A, ROBO2, PIWIL2, PTPRR, CTNND2, LDB2, FRY, LIPA, SYNE2, PHEX, PREX2, PPP3CA, GRIP1, NIPBL, BMPER, CHL1, SCMH1, PIP4K2A, PLCE1, SVEP1, DIP2B, PRKG1, TP63, SOX5, GRID2, PCYT1B, DCC, TRPC4, ATRX, TCF12, TET2, ANO6, WDR72, MMP16, NRG3, PALLD, TNMD, CDH13, RBM20, EPHA3, COL15A1, ARHGEF28, CEP85L, GREB1L, NRXN3, PCDH15, NEDD9, ATP2C1, HDAC9, PCDH17, RAD54B, SCEL, GRIN2A, WRN, CALD1, KRIT1, GPC3, SLIT3, CSMD3, CLASP1, SNCA, GRXCR1, NEGR1, PLEKHA5, KLHL1, CNOT4, TF, PTPRB, CDK6, DAB1, GRIN3A, FMNL2, NEDD4, UST, CNTN1, MNAT1, SMAP1, ADGRL3, FBN1
GO:0033036~macromolecule localization	0.034117922	47	DPP10, RAB3C, MCM9, RAP1GDS1, ITSN1, ATP2C1, GPHN, TM9SF3, PPP3CA, GRIP1, WRN, NIPBL, GRIN2A, LIPC, SCFD2, GPC3, BBS9, PIP4K2A, STXBP5, GPC5, CACNG3, TBC1D22A, CNTLN, CADPS2, ATP8B4, GRID2, CEP135, VPS13C, PRKCE, LRBA, VPS13D, ATRX, EXOC6B, WDR72, FRMD4A, PEX1, PARD3B, VAMP7, NBEA, NEDD4, AKAP9, MAPRE2, EPHA3, ABCG2, FBN1, RAPGEF4
**Term**	**p-value**	**Number of genes**	**Genes**
GO:0051716~cellular response to stimulus	0.035458023	101	SEMA5A, ERO1A, DGKB, CTNND2, ITSN1, ZBTB20, PREX2, BMPER, PIP4K2A, PLCE1, SVEP1, RGS6, TP63, KCNH1, SOX5, RGS7, ANKS1B, UNC13C, CASR, PRKCE, CACNA2D1, LEMD3, TBL1XR1, AKAP9, COL4A6, ELMO1, COL4A5, EPHA3, ARHGEF28, STK39, UBR1, PLD1, FHIT, GRIN2A, WDR70, XRCC6, GRIN2B, GPR137C, TF, CDK6, GRIN3A, TMEM117, GNAQ, CNTN1, GNB4, MNAT1, CDK14, FBN1, ROBO2, PTPRR, DOCK4, CAB39L, MCM9, DOCK8, PIK3C2G, GRM1, PPP3CA, NIPBL, NETO1, MECOM, GRM8, SPIN1, EPG5, DLGAP1, PRKG1, RALGPS1, VAV3, GRID2, PDE4D, VPS13C, ATRX, ANO6, ITFG1, NRG3, BANK1, TNMD, TFEC, CDH13, MAPRE2, RBMS3, RAPGEF4, COLEC12, NEDD9, ATP2C1, HDAC9, RAD54B, SCEL, WRN, MGAT5, GPC3, GPC5, SLIT3, UGGT2, CACNG3, SNCA, USP25, PLCL1, GABRA3, NEDD4, ADGRL3
GO:0048585~negative regulation of response to stimulus	0.042884713	28	SEMA5A, ROBO2, PTPRR, UBR1, SAMSN1, PPP3CA, BMPER, MECOM, GPC3, PIP4K2A, SLIT3, PRKG1, RGS6, TP63, CLASP1, RGS7, SNCA, USP25, GRID2, VPS13C, PDE4D, LEMD3, CDK6, GRIN3A, BANK1, TNMD, FBN1, RBMS3
GO:0048583~regulation of response to stimulus	0.043573169	59	SEMA5A, ROBO2, PTPRR, CTNND2, GRM1, PPP3CA, BMPER, NETO1, MECOM, SPIN1, PIP4K2A, EPG5, PLCE1, DLGAP1, PRKG1, RGS6, TP63, RALGPS1, RGS7, VAV3, GRID2, PRKCE, PDE4D, VPS13C, ANO6, LEMD3, VAMP7, TBL1XR1, BANK1, TNMD, AKAP9, CDH13, MAPRE2, RBMS3, COLEC12, CAMK2D, ARHGEF28, STK39, UBR1, ATP2C1, SAMSN1, SCEL, WDR70, MGAT5, GPC3, SLIT3, GPC5, CACNG3, CLASP1, SNCA, USP25, XRCC6, GPR137C, CDK6, GRIN3A, NEDD4, GNAQ, CDK14, FBN1
GO:0044456~synapse part	5.07E-11	34	RAB3C, CTTNBP2, CTNND2, ITSN1, GRIK1, GRM1, GPHN, PCDH17, PPP3CA, GRIN2A, SV2C, NETO1, STXBP5, KCNN2, DLGAP1, CACNG3, KCNH1, SNCA, UNC13C, CADPS2, CASR, GRID2, DCC, GABRA3, PLEKHA5, SORCS3,

			GRIN2B, DNM3, GRIN3A, NEDD4, AKAP9, CNTN1, UTRN
GO:0098794~postsynapse	7.62E-11	26	CTTNBP2, CTNND2, GRIK1, GRM1, GPHN, PCDH17, PPP3CA, GRIN2A, NETO1, KCNN2, DLGAP1, CACNG3, KCNH1, SNCA, GRID2, DCC, GABRA3, PLEKHA5, SORCS3, GRIN2B, DNM3, GRIN3A, NEDD4, CNTN1, UTRN
GO:0097060~synaptic membrane	3.54E-10	20	UNC13C, GRID2, DCC, GABRA3, GRIK1, SORCS3, GRIN2B, GRM1, GPHN, PCDH17, DNM3, GRIN2A, GRIN3A, NETO1, AKAP9, CNTN1, UTRN, CACNG3, KCNH1
GO:0097458~neuron part	1.63E-09	44	RAB3C, DOCK4, CAMK2D, KLHL14, KCNC2, CTTNBP2, CTNND2, ITSN1, PCDH15, GRM1, GPHN, PCDH17, PPP3CA, GRIP1, GRIN2A, SV2C, CHL1, STXBP5, KCNN2, DIP2B, CACNG3, IMPG1, KCNH1, SNCA, UNC13C, CADPS2, GRXCR1, CASR, GRID2, DCC, GABRA3, KLHL1, SLC6A11, DNM3, GRIN3A, VAMP7, PALLD, NEDD4, GNAQ, CNTN1, CDH13, EPHA3, ADGRL3
GO:0098978~glutamatergic synapse	2.32E-08	17	CADPS2, GRID2, DGKB, PPM1H, PLEKHA5, GRM1, PCDH17, PPP3CA, GRIP1, GRIN2A, GRIN3A, NRG3, NETO1, AKAP9, DLGAP1, CACNG3
GO:0099634~postsynaptic specialization membrane	2.90E-08	11	GRIN2A, GRID2, NETO1, DCC, SORCS3, GRIN2B, CACNG3, GPHN, GRM1, KCNH1
GO:1902495~transmembrane transporter complex	3.07E-08	19	DPP10, GRID2, KCNC2, TRPC4, PDE4D, CACNA2D1, GABRA3, ANO6, GRIN2B, GRIN2A, OLFM3, GRIN3A, AKAP9, CACHD1, MICU3, STXBP5, CACNG3, KCNH1

**Term**	**p-value**	**Number of genes**	**Genes**
GO:1990351~transporter complex	6.43E-08	19	DPP10, GRID2, KCNC2, TRPC4, PDE4D, CACNA2D1, GABRA3, ANO6, GRIN2B, GRIN2A, OLFM3, GRIN3A, AKAP9, CACHD1, MICU3, STXBP5, CACNG3, KCNH1
GO:0099572~postsynaptic specialization	3.56E-07	15	GRID2, DCC, CTNND2, PLEKHA5, SORCS3, GRIN2B, GRM1, GPHN, DNM3, GRIN2A, NETO1, DLGAP1, CACNG3, KCNH1
GO:0098936~intrinsic component of postsynaptic membrane	1.14E-06	9	GRIN2A, GRID2, NETO1, DCC, CNTN1, SORCS3, CACNG3, PCDH17
GO:0044459~plasma membrane part	1.48E-06	56	DPP10, KCNC2, DOCK8, GRIK1, GPHN, GRM1, PPP3CA, NETO1, GRM8, SLC16A7, KCNH1, VAV3, UNC13C, GRID2, CASR, KCNH7, DCC, TRPC4, SLC2A13, CACNA2D1, SLC6A11, SORCS3, TMC5, PARD3B, DNM3, OLFM3, MMP16, AKAP9, CDH13, UTRN, EPHA3, ABCG2, SLC20A2, PCDH15, STK39, PLD1, PCDH17, GRIN2A, GPC3, STXBP5, GPC5, CACNG3, JAM2, SLC38A4, GABRA3, GRIN2B, ENOX1, TF, GRIN3A, GNAQ, GNB4, CACHD1, CNTN1, SLC26A7, ADGRL3
GO:0098590~plasma membrane region	2.32E-06	29	KCNC2, STK39, GRIK1, PLD1, GRM1, GPHN, PCDH17, GRIN2A, NETO1, CACNG3, KCNH1, UNC13C, GRID2, DCC, TRPC4, GABRA3, SORCS3, GRIN2B, PARD3B, DNM3, TF, GRIN3A, AKAP9, CNTN1, SLC26A7, CDH13, UTRN, ABCG2
GO:0098984~neuron to neuron synapse	4.34E-06	13	DNM3, GRID2, GRIN2A, NETO1, DCC, CTNND2, PLEKHA5, SORCS3, GRIN2B, CACNG3, GRM1, KCNH1

GO:0042995~cell projection	9.55E-06	45	DOCK4, CAMK2D, KLHL14, KCNC2, CTTNBP2, ITSN1, PCDH15, GRM1, GPHN, PPP3CA, GRIP1, GRIN2A, SV2C, CHL1, BBS9, PLCE1, KCNN2, DIP2B, IMPG1, KCNH1, SNCA, UNC13C, GRXCR1, CASR, GRID2, DCC, SLC2A13, GABRA3, KLHL1, MICAL3, SLC6A11, FRMD4B, DNM3, CDK6, GRIN3A, VAMP7, PALLD, NEDD4, GNAQ, CDH13, UTRN, EPHA3, ABCG2, ADGRL3
GO:0098948~intrinsic component of postsynaptic specialization membrane	1.79E-05	7	GRIN2A, GRID2, NETO1, DCC, SORCS3, CACNG3
**Term**	**p-value**	**Number of genes**	**Genes**
GO:0043235~receptor complex	8.64E-04	14	GRID2, GABRA3, GRIN2B, LRP1B, GRM1, TF, PTPRB, GRIN2A, OLFM3, GRIN3A, STXBP5, CACNG3, EPHA3
GO:0098793~presynapse	8.64E-04	14	RAB3C, UNC13C, CADPS2, CASR, ITSN1, PCDH17, DNM3, GRIN2A, SV2C, CNTN1, STXBP5, SNCA, KCNH1
GO:0070161~anchoring junction	0.001848958	15	FOCAD, CTNND2, GABRA3, FRMD4A, FRMD4B, LPP, PARD3B, PPP3CA, VAMP7, PALLD, CTNNA3, MAPRE2, CLASP1, SNCA, KCNH1
GO:0071944~cell periphery	0.002678317	111	RAB3C, DGKB, CTNND2, FRY, PREX2, GRIP1, LIPH, PIP4K2A, PLCE1, SLC16A7, KCNH1, RGS7, UNC13C, CASR, KCNH7, PRKCE, CACNA2D1, SLC6A11, OLFM3, AKAP9, SLC9A9, ELMO1, UTRN, EPHA3, ABCG2, PARM1, CTTNBP2, SLC41A2, STK39, PCDH15, PLD1, FHIT, PCDH17, GRIN2A, CALD1, SCFD2, STXBP5, KCNN2, SLC38A4, ATP8B4, MICAL3, EXOC6B, GRIN2B, TF, GRIN3A, NBEA, TMEM117, GNAQ, CNTN1, GNB4, SLC26A7, CDK14, DPP10, PTPRR, DOCK4, SLC44A1, KCNC2, DOCK8, GRIK1, PIK3C2G, LDB2, GRM1, GPHN, PHEX, PPP3CA, NETO1, CHL1, GRM8, PRKG1, VAV3, GRID2, DCC, TRPC4, PDE4D, SLC2A13, ANO6, TMC5, SORCS3, ITFG1, PARD3B, DNM3, VAMP7, MMP16, PALLD, CDH13, CAMK2D, SLC20A2, NRXN3, NEDD9, ATP2C1, GPC3, KRIT1, GPC5, CSMD3, CACNG3, CORIN, CLASP1, JAM2, SNCA, NEGR1, EGF, PLCL1, GABRA3, LHFPL3, DYTN, ENOX1, NEDD4,

			CACHD1, FILIP1, ADGRL3
GO:0031012~extracellular matrix	0.004388014	11	COL28A1, COLEC12, ADAMTSL1, COL15A1, MMP16, BMPER, COL4A6, COL4A5, TINAG, IMPG1, FBN1
GO:0098685~Schaffer collateral - CA1 synapse	0.006294513	5	PPP3CA, DCC, DGKB, CACNG3, GRM1

**Term**	**p-value**	**Number of genes**	**Genes**
GO:0005886~plasma membrane	0.006674866	107	RAB3C, DGKB, CTNND2, PREX2, GRIP1, LIPH, PIP4K2A, PLCE1, SLC16A7, KCNH1, RGS7, UNC13C, CASR, KCNH7, PRKCE, CACNA2D1, SLC6A11, OLFM3, AKAP9, SLC9A9, ELMO1, UTRN, EPHA3, ABCG2, PARM1, SLC41A2, STK39, PCDH15, PLD1, FHIT, PCDH17, GRIN2A, CALD1, SCFD2, STXBP5, KCNN2, SLC38A4, ATP8B4, MICAL3, GRIN2B, TF, GRIN3A, NBEA, TMEM117, GNAQ, CNTN1, GNB4, SLC26A7, CDK14, DPP10, PTPRR, DOCK4, SLC44A1, KCNC2, DOCK8, GRIK1, PIK3C2G, LDB2, GRM1, GPHN, PHEX, PPP3CA, NETO1, CHL1, GRM8, PRKG1, VAV3, GRID2, DCC, TRPC4, PDE4D, SLC2A13, ANO6, TMC5, SORCS3, ITFG1, PARD3B, DNM3, VAMP7, MMP16, PALLD, CDH13, CAMK2D, SLC20A2, NRXN3, NEDD9, ATP2C1, GPC3, KRIT1, GPC5, CSMD3, CACNG3, CORIN, JAM2, SNCA, NEGR1, EGF, PLCL1, GABRA3, LHFPL3, DYTN, ENOX1, NEDD4, CACHD1, FILIP1, ADGRL3
GO:0044297~cell body	0.007062051	10	GRIN3A, KLHL14, KCNC2, CTNND2, SLC2A13, KLHL1, KCNN2, DIP2B, SNCA, KCNH1
GO:0019898~extrinsic component of membrane	0.007238142	10	COQ3, TF, VPS13C, GNAQ, VPS13D, GNB4, CDH13, PIK3C2G, STXBP5, RGS6
GO:0012505~endomemb	0.01131271	58	ERO1A, RAB3C, DOCK4, ITSN1, SYNE2, PHEX, CCDC91, SPIN1, PRKG1, TP63, KCNH1, UNC13C, CHST9, PCYT1B,

rane system			PRKCE, VPS13C, CACNA2D1, SORCS1, WDR72, CDKAL1, LEMD3, CPED1, OLFM3, VAMP7, AKAP9, SLC9A9, VOPP1, EPHA3, CDS1, CAMK2D, PARM1, KLHL14, RNF180, RAP1GDS1, ATP2C1, PLD1, GRIN2A, SV2C, SCFD2, MGAT5, CLVS2, STXBP5, ST8SIA6, UGGT2, CLASP1, SNCA, USP25, ATP8B4, B3GAT2, TF, NBEA, NEDD4, TMEM117, UST, TMCC3, SLC26A7, GALNTL6
GO:0044420~extracellular matrix component	0.011755793	6	COL28A1, COL15A1, COL4A6, COL4A5, TINAG, FBN1

Term	p-value	Number of genes	Genes
GO:0044424~intracellular part	0.030195261	189	ERO1A, RAB3C, CTNND2, PREP, ZBTB20, FRY, LIPA, CCDC91, CSPP1, RAVER2, SCMH1, PIP4K2A, PLCE1, SLC16A7, SVEP1, DIP2B, RGS6, TP63, KCNH1, SOX5, RGS7, ANKS1B, UNC13C, CADPS2, CDKL4, CEP135, UBE2E3, PRKCE, CACNA2D1, WDR72, CDKAL1, FRMD4A, FRMD4B, PPP4R3B, LEMD3, FOXP2, FOXP1, CPED1, INPP4B, OLFM3, TBL1XR1, AKAP9, SLC9A9, ELMO1, VOPP1, UTRN, EPHA3, ABCG2, CDS1, PARM1, KLHL14, CTTNBP2, ARHGEF28, RNF180, CEP85L, TSHZ2, CWF19L2, STK39, UBR1, PLD1, LPP, SAMSN1, FHIT, GRIN2A, WDR70, CALD1, SCFD2, TASP1, STXBP5, ST8SIA6, CNTLN, ATP8B4, XRCC6, LRBA, MICAL3, EXOC6B, MYO5A, AMMECR1, PHC3, GPR137C, CNOT4, PKIB, COQ3, TF, CDK6, DAB1, NBEA, KIF4A, TMEM117, GNAQ, UST, GNB4, TMCC3, SLC26A7, GALNTL6, MNAT1, CDK14, PIWIL2, PTPRR, DOCK4, SLC44A1, MCM9, DOCK8, PIK3C2G, LDB2, GRM1, SYNE2, GPHN, PHEX, PPP3CA, NIPBL, MECOM, SPIN1, BBS9, EPG5, ZNF644, RNF150, TINAG, PRKG1, CHST9, MYBPC1, PCYT1B, TRPC4, PDE4D, VPS13C, TCF12, ATRX, TET2, ANO6, SORCS1, HAUS6, PEX1, NAALADL2, PARD3B, DNM3, VAMP7, SETBP1, PCCA, BANK1, PALLD, DPYD, TFEC, CDH13, RBMS1, RBM20, MICU3, MAPRE2, RBKS, RBMS3, CREB5, RAPGEF4, CAMK2D, RAP1GDS1, PPM1H, NEDD9, HSD17B11, ATP2C1, HDAC9, NOL4, RAD54B, SCEL, WRN, SV2C, MGAT5,

GO:0005622~intracellular	0.030195261	189	GPC3, CLVS2, KRIT1, CTNNA3, UGGT2, CORIN, CLASP1, RUNX1T1, SNCA, FARP1, USP25, B3GAT2, KLHL1, PLEKHA5, LSAMP, YLPM1, CENPE, FMNL2, XPNPEP1, CENPI, NEDD4, EIF3H, FILIP1, SMAP1 ERO1A, RAB3C, CTNND2, PREP, ZBTB20, FRY, LIPA, CCDC91, CSPP1, RAVER2, SCMH1, PIP4K2A, PLCE1, SLC16A7, SVEP1, DIP2B, RGS6, TP63, KCNH1, SOX5, RGS7, ANKS1B, UNC13C, CADPS2, CDKL4, CEP135, UBE2E3, PRKCE, CACNA2D1, WDR72, CDKAL1, FRMD4A, FRMD4B, PPP4R3B, LEMD3, FOXP2, FOXP1, CPED1, INPP4B, OLFM3, TBL1XR1, AKAP9, SLC9A9, ELMO1, VOPP1, UTRN, EPHA3, ABCG2, CDS1, PARM1, KLHL14, CTTNBP2, ARHGEF28, RNF180, CEP85L, TSHZ2, CWF19L2, STK39, UBR1, PLD1, LPP, SAMSN1, FHIT, GRIN2A, WDR70, CALD1, SCFD2, TASP1, STXBP5, ST8SIA6, CNTLN, ATP8B4, XRCC6, LRBA, MICAL3, EXOC6B, MYO5A, AMMECR1, PHC3, GPR137C, CNOT4, PKIB, COQ3, TF, CDK6, DAB1, NBEA, KIF4A, TMEM117, GNAQ, UST, GNB4, TMCC3, SLC26A7, GALNTL6, MNAT1, CDK14, PIWIL2, PTPRR, DOCK4, SLC44A1, MCM9, DOCK8, PIK3C2G, LDB2, GRM1, SYNE2, GPHN, PHEX, PPP3CA, NIPBL, MECOM, SPIN1, BBS9, EPG5, ZNF644, RNF150, TINAG, PRKG1, CHST9, MYBPC1, PCYT1B, TRPC4, PDE4D, VPS13C, TCF12, ATRX, TET2, ANO6, SORCS1, HAUS6, PEX1, NAALADL2, PARD3B, DNM3, VAMP7, SETBP1, PCCA, BANK1, PALLD, DPYD, TFEC, CDH13, RBMS1, RBM20, MICU3, MAPRE2, RBKS, RBMS3, CREB5, RAPGEF4, CAMK2D, RAP1GDS1, PPM1H, NEDD9, HSD17B11, ATP2C1, HDAC9, NOL4, RAD54B, SCEL, WRN, SV2C, MGAT5, GPC3, CLVS2, KRIT1, CTNNA3, UGGT2, CORIN, CLASP1, RUNX1T1, SNCA, FARP1, USP25, B3GAT2, KLHL1, PLEKHA5, LSAMP, YLPM1, CENPE, FMNL2, XPNPEP1, CENPI, NEDD4, EIF3H, FILIP1, SMAP1
**Term**	**p-value**	**Number of genes**	**Genes**
GO:0031594~neuromuscular junction	0.039114937	4	UNC13C, COL4A5, UTRN, PDZRN3
GO:0031252~cell leading edge	0.039178025	9	VAMP7, CDK6, KCNC2, PALLD, DOCK8, GABRA3, PLCE1, LDB2, FRMD4B
GO:0005515~protein binding	8.36E-06	111	SEMA5A, RAB3C, CTNND2, ITSN1, GRIP1, LIPC, SCMH1, PIP4K2A, PLCE1, DIP2B, TBC1D22A, TP63, KCNH1, RGS7, ANKS1B, UNC13C, CASR, CEP135, PRKCE, FRMD4A, FOXP1, TBL1XR1, AKAP9, ELMO1, UTRN, ABCG2, RNF180, SAMSN1, FHIT, WDR70, CALD1, SCFD2, TASP1, STXBP5, KCNN2, CNTLN, XRCC6, LRBA, MICAL3, MYO5A, PHC3,

			PTPRB, DIAPH2, TF, CDK6, GRIN3A, NBEA, KIF4A, GNAQ, GNB4, TMCC3, MNAT1, CDK14, FBN1, DPP10, ROBO2, DOCK4, MCM9, LDB2, SYNE2, GPHN, PPP3CA, NIPBL, NETO1, CHL1, SPIN1, ZNF644, PRKG1, GRID2, MYBPC1, TRPC4, PDE4D, SLC2A13, TCF12, ATRX, ANO6, HAUS6, PEX1, DNM3, VAMP7, NRG3, PCCA, BANK1, PALLD, DPYD, TFEC, CDH13, MAPRE2, RBKS, RAPGEF4, CAMK2D, PPM1H, NEDD9, HDAC9, WRN, KRIT1, SLIT3, CTNNA3, UGGT2, CLASP1, JAM2, SNCA, FARP1, USP25, PLCL1, CENPE, FMNL2, XPNPEP1, NEDD4, SMAP1
GO:0005085~guanyl-nucleotide exchange factor activity	8.94E-06	13	VAV3, FARP1, DOCK4, ARHGEF28, DOCK8, RAP1GDS1, ITSN1, PREX2, ELMO1, PLCE1, MCF2L2, RALGPS1, RAPGEF4
GO:0043167~ion binding	1.15E-04	95	ERO1A, HBS1L, RAB3C, DGKB, MCM9, ITSN1, PIK3C2G, GPHN, CLYBL, PHEX, PPP3CA, LIPC, LIPH, PIP4K2A, SVEP1, RNF150, PRKG1, TP63, KCNH1, VAV3, UNC13C, CADPS2, CASR, PCYT1B, CDKL4, TRPC4, PRKCE, PDE4D, UBE2E3, ATRX, TET2, CDKAL1, PEX1, FOXP2, FOXP1, PARD3B, DNM3, MMP16, PCCA, DPYD, CDH13, RBM20, MICU3, TNIK, UTRN, EPHA3, RBKS, ABCG2, RAPGEF4, COLEC12, CAMK2D, RNF180, TSHZ2, PCDH15, STK39, UBR1, ATP2C1, PLD1, LPP, HDAC9, PCDH17, RAD54B, SCEL, SNX29, WRN, MGAT5, KRIT1, CLVS2, SLIT3, IMPG1, RUNX1T1, SNCA, ATP8B4, XRCC6, B3GAT2, PLCL1, MICAL3, PLEKHA5, MYO5A, PHC3, GRIN2B, LRP1B, DYTN, CNOT4, CENPE, TF, CDK6, GRIN3A, XPNPEP1, KIF4A, MNAT1, CDK14, SMAP1, ADGRL3, FBN1

**Term**	**p-value**	**Number of genes**	**Genes**
GO:0022857~transmembrane transporter activity	2.59E-04	29	SLC44A1, SLC20A2, KCNC2, SLC41A2, GRIK1, ATP2C1, GRIN2A, SV2C, SLC16A7, KCNN2, CACNG3, KCNH1, SLC38A4, GRID2, KCNH7, TRPC4, CACNA2D1, SLC2A13, GABRA3, SLC6A11, ANO6, TMC5, GRIN2B, GRIN3A, SLC9A9, CACHD1, SLC26A7, ABCG2
GO:0022892~substrate-specific transporter activity	7.44E-04	27	SLC44A1, KCNC2, SLC41A2, GRIK1, ATP2C1, GRIN2A, SLC16A7, KCNN2, CACNG3, KCNH1, SLC38A4, GRID2, KCNH7, TRPC4, CACNA2D1, SLC2A13, GABRA3, SLC6A11, ANO6, TMC5, GRIN2B, GRIN3A, SLC9A9, CACHD1, SLC26A7, ABCG2
GO:0036094~small molecule binding	0.007437261	45	ERO1A, HBS1L, RAB3C, CAMK2D, DGKB, MCM9, STK39, PIK3C2G, UBR1, ATP2C1, FHIT, GPHN, RAD54B, WRN, PIP4K2A, PRKG1, SNCA, CASR, ATP8B4, CDKL4, XRCC6, TRPC4, UBE2E3, PLCL1, PRKCE, PDE4D, ATRX, MICAL3,

			MYO5A, PEX1, GRIN2B, DNM3, CENPE, CDK6, GRIN3A, PCCA, GNAQ, KIF4A, DPYD, TNIK, CDK14, EPHA3, RBKS, ABCG2, RAPGEF4
GO:0005201~extracellular matrix structural constituent	0.007851658	5	COL28A1, COL15A1, COL4A6, COL4A5, FBN1
GO:0044877~macromolecular complex binding	0.014569552	21	COLEC12, PIWIL2, XRCC6, MCM9, MICAL3, MYO5A, PEX1, SYNE2, CENPE, WRN, NIPBL, FMNL2, GNAQ, GNB4, CDH13, CTNNA3, UTRN, CLASP1, JAM2, FBN1, SNCA
GO:0097367~carbohydrate derivative binding	0.017009545	39	HBS1L, RAB3C, CAMK2D, DGKB, MCM9, STK39, PIK3C2G, ATP2C1, GPHN, RAD54B, WRN, LIPC, LIPH, PIP4K2A, SLIT3, IMPG1, PRKG1, CLASP1, ATP8B4, CDKL4, XRCC6, UBE2E3, PRKCE, PDE4D, ATRX, MYO5A, PEX1, DNM3, CENPE, CDK6, PCCA, KIF4A, TNIK, CDK14, EPHA3, RBKS, ABCG2, FBN1, RAPGEF4

Table S4. KEGG enrichment results of common differential methylation genes in spleen and liver groups

Term	*p*-value	Number of genes	Genes
bta04724:Glutamatergic synapse	1.24E-06	11	PPP3CA, GRIN2A, GRIN3A, GNAQ, GRM8, GNB4, GRIK1, DLGAP1, PLD1, GRIN2B, GRM1
bta05031:Amphetamine addiction	1.62E-03	6	PPP3CA, CAMK2D, GRIN2A, GRIN3A, GRIN2B, CREB5
bta04720:Long-term potentiation	1.62E-03	6	PPP3CA, CAMK2D, GRIN2A, GNAQ, GRIN2B, GRM1
bta04024:cAMP signaling pathway	3.19E-03	10	VAV3, CAMK2D, GRIN2A, GRIN3A, PDE4D, PLCE1, PLD1, GRIN2B, RAPGEF4, CREB5
bta04015:Rap1 signaling pathway	5.52E-03	9	VAV3, GRIN2A, DOCK4, EGF, GNAQ, KRIT1, PLCE1, GRIN2B, RAPGEF4
bta04728:Dopaminergic synapse	6.38E-03	7	PPP3CA, CAMK2D, GRIN2A, GNAQ, GNB4, GRIN2B, CREB5
bta04360:Axon guidance	6.82E-03	8	ROBO2, SEMA5A, PPP3CA, CAMK2D, DCC, TRPC4, SLIT3, EPHA3
bta04070:Phosphatidylinositol signaling system	7.05E-03	6	CDS1, INPP4B, DGKB, PIP4K2A, PLCE1, PIK3C2G
bta04713:Circadian entrainment	8.33E-03	6	CAMK2D, GRIN2A, GNAQ, GNB4, GRIN2B, PRKG1

bta04928:Parathyroid hormone synthesis, secretion and action	9.77E-03	6	CASR, MMP16, GNAQ, PDE4D, PLD1, CREB5
bta04072:Phospholipase D signaling pathway	1.19E-02	7	DNM3, EGF, DGKB, GRM8, PLD1, GRM1, RAPGEF4
bta05033:Nicotine addiction	1.32E-02	4	GRIN2A, GRIN3A, GABRA3, GRIN2B
bta04911:Insulin secretion	2.12E-02	5	CAMK2D, GNAQ, KCNN2, RAPGEF4, CREB5
bta05030:Cocaine addiction	2.15E-02	4	GRIN2A, GRIN3A, GRIN2B, CREB5
bta04727:GABAergic synapse	2.64E-02	5	PLCL1, GABRA3, GNB4, SLC6A11, GPHN
bta05231:Choline metabolism in cancer	3.45E-02	5	PCYT1B, SLC44A1, EGF, DGKB, PLD1
bta05017:Spinocerebellar ataxia	3.51E-02	6	GRIN2A, GRIN3A, DAB1, GNAQ, GRIN2B, GRM1
bta04261:Adrenergic signaling in cardiomyocytes	3.88E-02	6	CAMK2D, CACNA2D1, GNAQ, CACNG3, RAPGEF4, CREB5
bta04922:Glucagon signaling pathway	3.91E-02	5	PPP3CA, CAMK2D, GNAQ, PPP4R3B, CREB5
bta04730:Long-term depression	3.99E-02	4	GRID2, GNAQ, PRKG1, GRM1

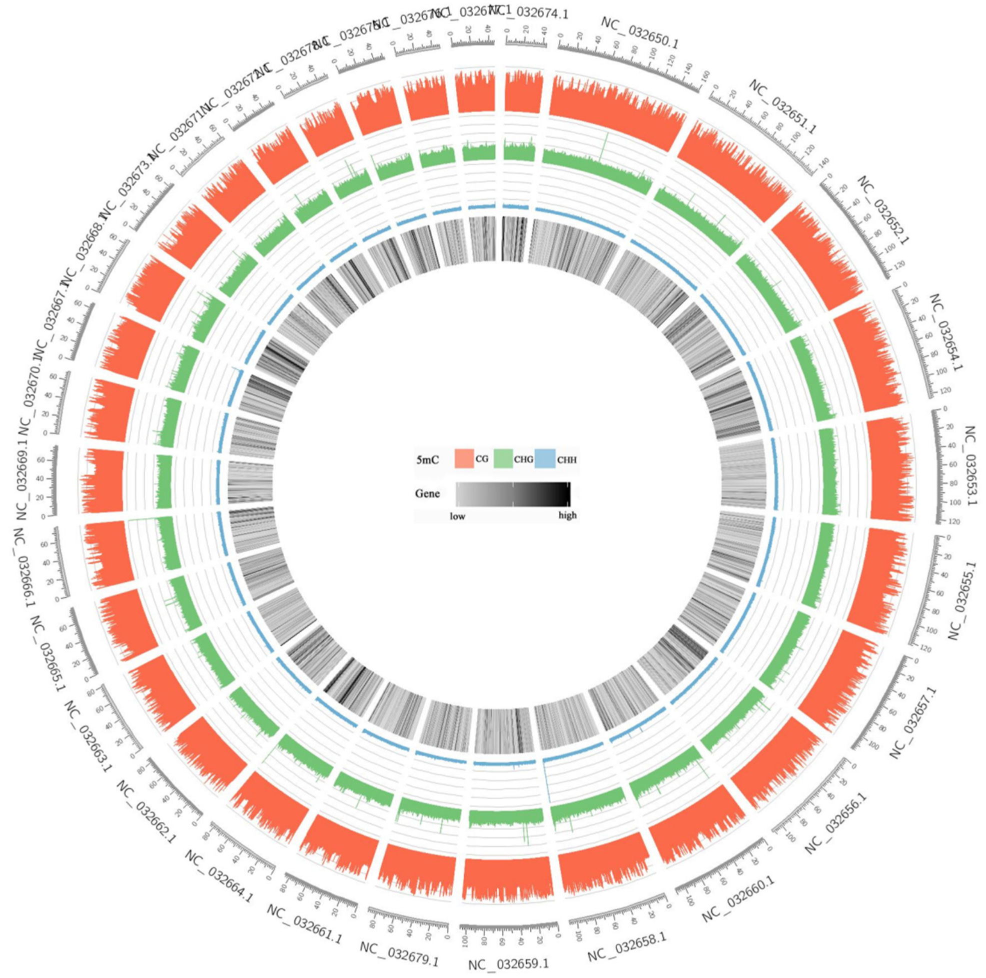

Figure S1. PBI15 whole-genome DNA methylation patterns

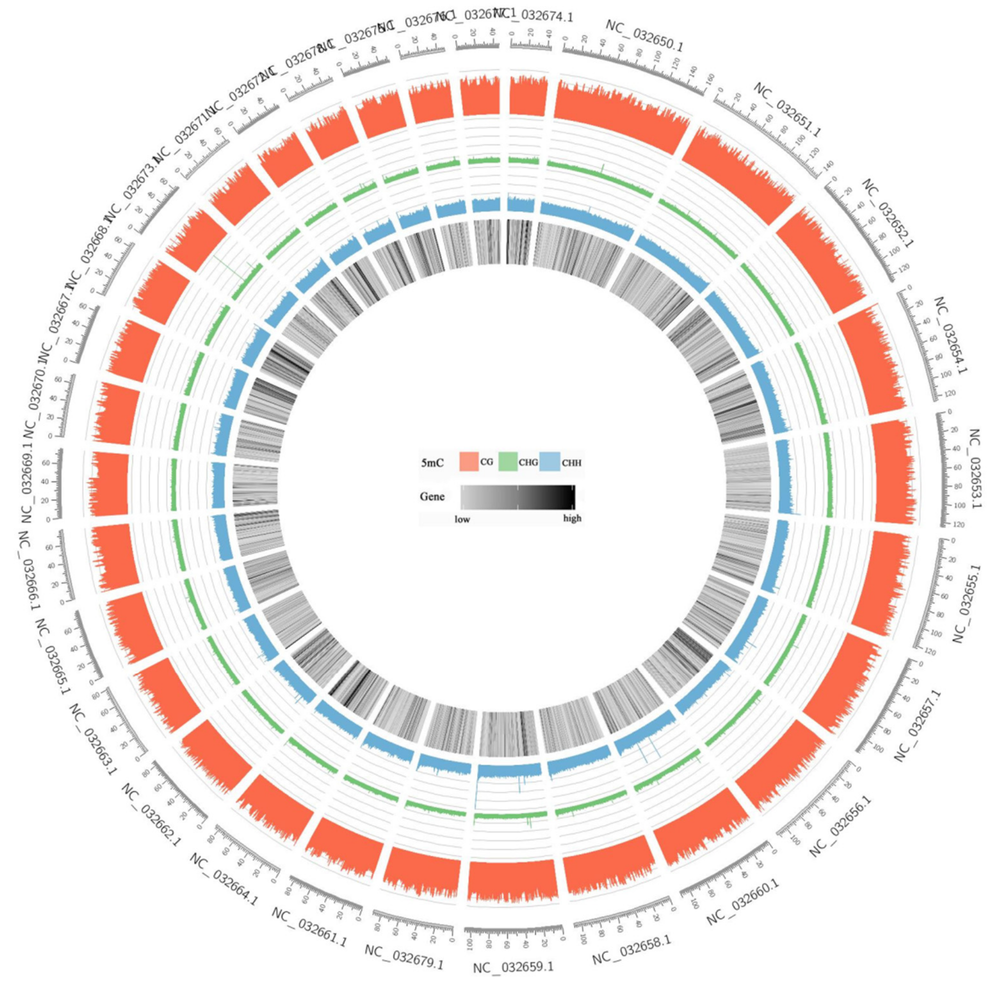

Figure S2. PBI52 whole-genome DNA methylation patterns

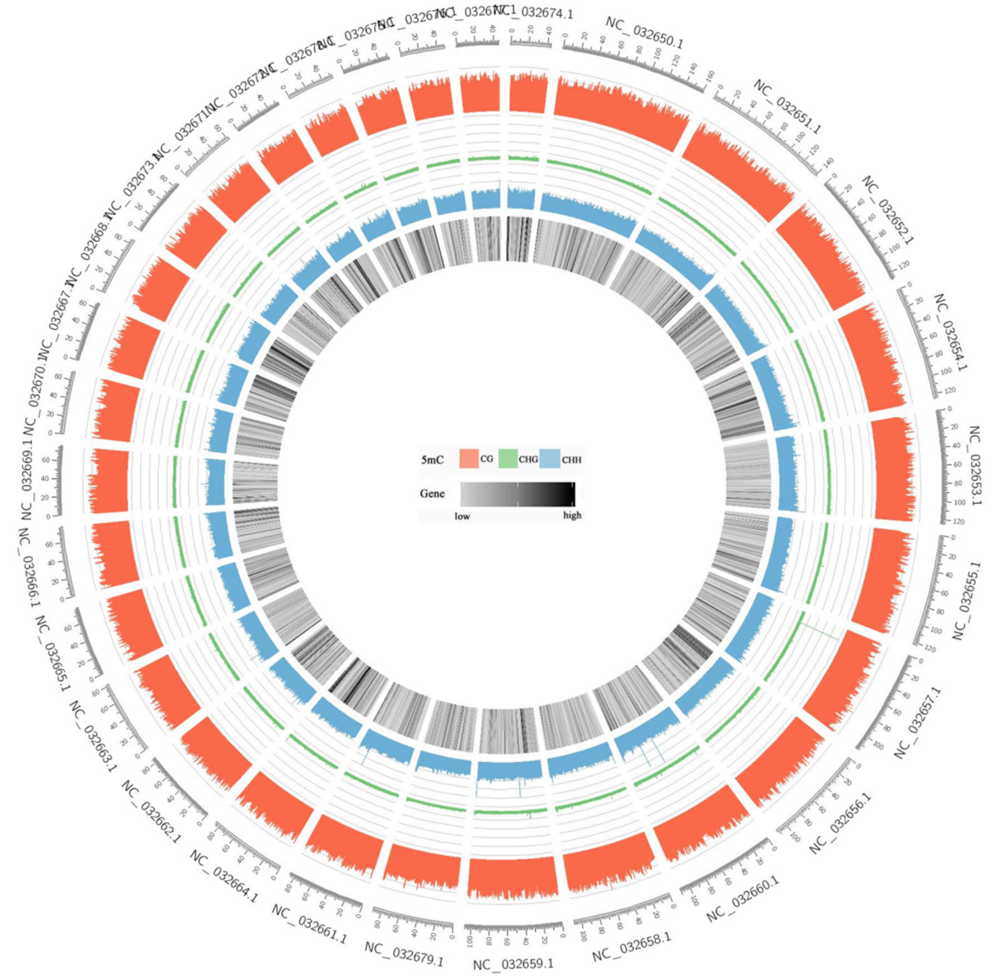

Figure S3. PBT24 whole-genome DNA methylation patterns

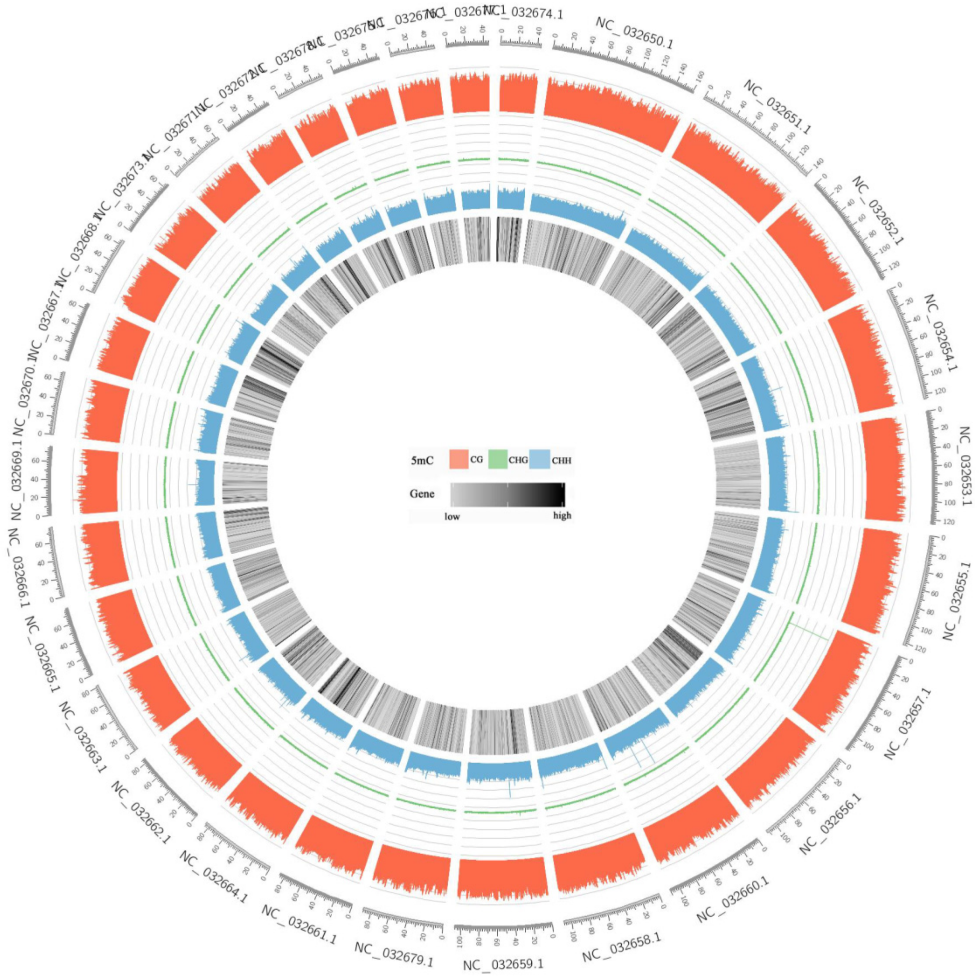

Figure S4. PBT47 whole-genome DNA methylation patterns

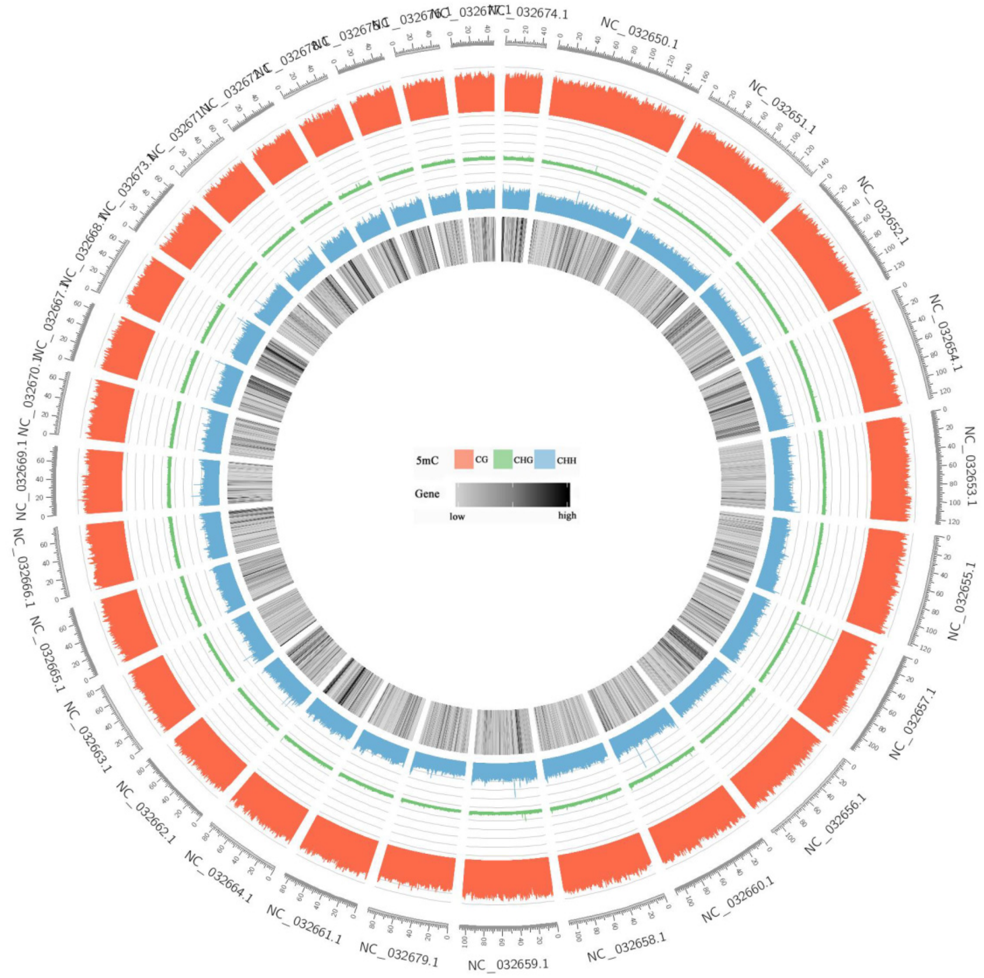

Figure S5. PBT57 whole-genome DNA methylation patterns

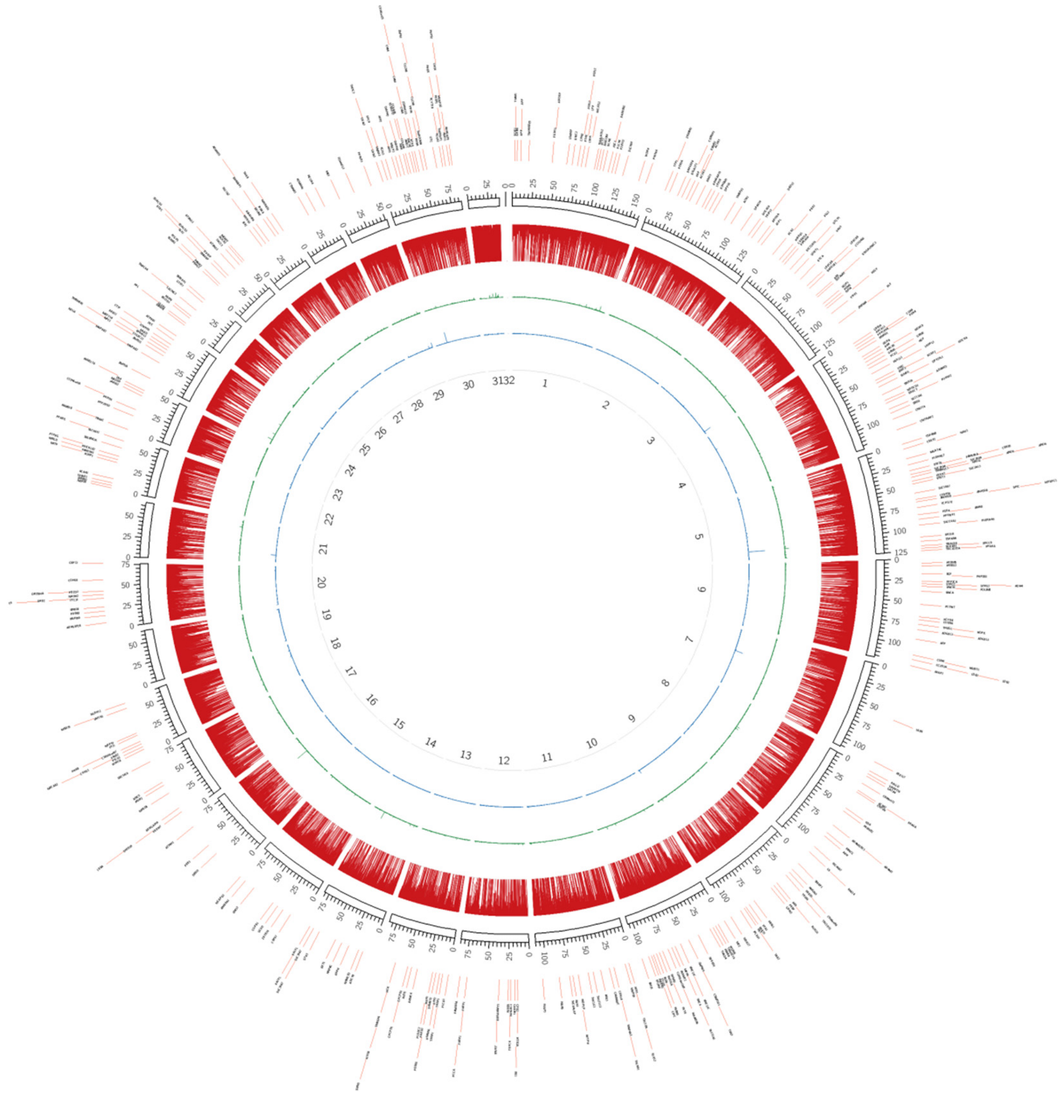

Figure S6. GBI52 whole-genome DNA methylation patterns

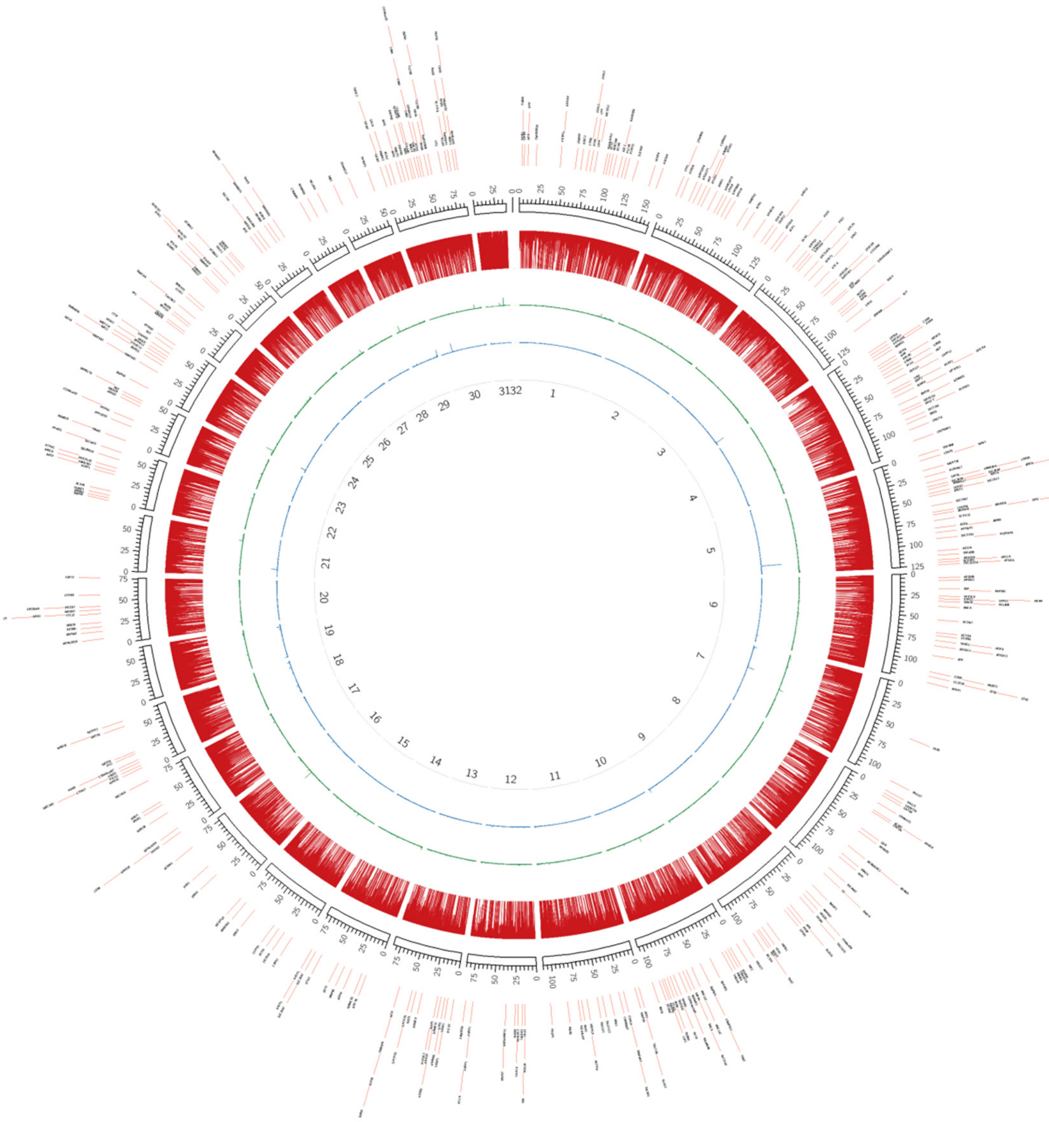

Figure S7. GBT47 whole-genome DNA methylation patterns

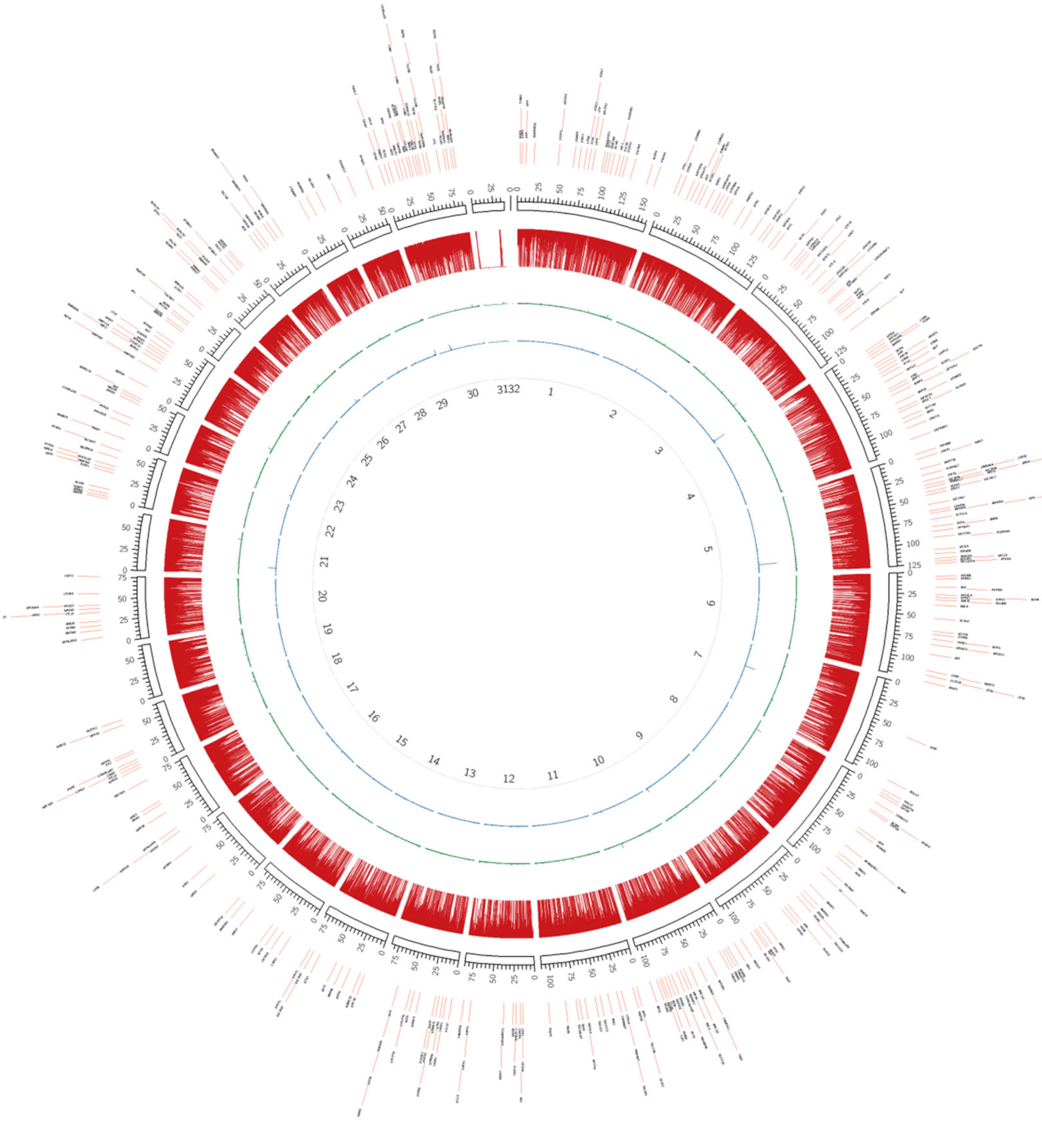

Figure S8. GBT57 whole-genome DNA methylation patterns
